# Supplementary material for: Are there sex differences in physiological parameters and reaction time responses to overload in firefighters?
Source: PLoS One. 2021 May 3;16(5):e0249559. doi: 10.1371/journal.pone.0249559 (PMC8092788; doi:10.1371/journal.pone.0249559)
Supplement: Attachment — Submitted filename: Response to Rev 2 1.2.2021 LG.docx [file pone.0249559.s002.docx]

PONE-D-20-30525
Are there sex differences in physiological parameters and reaction time responses to overload in firefighters?
PLOS ONE

We thank the Editor and the reviewers for their interesting and useful comments that helped enhancing the quality of our paper. We do hope that, thanks to the reviewers’ comments, we could successfully deal with the requested revisions, rendering the paper better readable. To facilitate the identification of the revisions, we have highlighted in red color all new or modified sentences according to the reviewers

Review Comments to the Author

Please use the space provided to explain your answers to the questions above. You may also include additional comments for the author, including concerns about dual publication, research ethics, or publication ethics. (Please upload your review as an attachment if it exceeds 20,000 characters)

Reviewer #1: Thank you for your response to my concerns. Regarding the reaction time data, this reviewer strongly feels that the authors need control data. I understand that the reaction time baseline value was taken prior to exercise, but what happens to the reaction time value if you repeat it three times without exercise in this same population? How variable is this measure? What happens to the reaction time if you were to repeat this test in a non-firefighter population? There needs to be additional control data to significantly strengthen this manuscript.

*Answer: To evaluate the RT, previous researches used FITLIGHT TrainerTM . For example, Zwierko et al. (2014) showed that non-athletes had longer RTs than athletes, Fischer et al. (2015) used this instrument in the United States Air Force, and Zurek et al. (2015) and Genovese et al. (2020) investigated the simple and complex RTs of athletes players who had undergone knee surgery and a rehabilitation program to assess their recovery.* *In addition, Reigal et al. (2019) studied the reliability of the FITLIGHT TrainerTM using the intraclass correlation index (IC), the standard error of measurement (SEM), and the minimal difference (MD).* *The results showed ICC_2;1_ = 0.92, SEM = 39.87, and MD = 110.51 ms for the simple task.* *For the complex task, they found ICC_2;1_ = 0.85, SEM = 63.50, and MD = 176.00 ms, which can be considered as adequate reliability indices.*

*To increase the clarity of text we changed as follow:*

“…………………

Reaction Time Test

The Fitlight Trainer ^®^ (FitLight Sports Corp, Ontario, Canada) was used to assed the RT to visual stimulus. It is a wireless reaction system, comprised of eight LED lights controlled by a tablet computer; a sensor reacts to proximity or touch and switches off the light. Previous study of Reigal et al. (2019) found adequate reliability indices of the Fitlight Trainer ^®^ for simple (ICC_2;1_ = 0.92, SEM = 39.87, and MD = 110.51 ms) and complex (ICC_2;1_ = 0.85, SEM = 63.50, and MD = 176.00 ms) task. Participants, standing upright with arms along the hips, at about 30 cm from the wall, centred to………….”

“Previously, our group [28] found that firefighters made significantly more errors than no firefighters in basal condition, where the RT required was high, underlining a lower level of cognitive function. We hypothesized that the “static” execution of the test did not reproduce the highly variable working conditions on where firefighters usually operate. Compared with our previous study [28], the new results demonstrated low value in both female and male recruits for MRT in all trial, but with a different trend for errors.”

Further, while the amended manuscript includes more of a rational for studying female firefighters (injury risk), the physiological relevance of a sex difference related to the slope of the line of the heart rate reserve, is unclear. What does a difference in slope indicate and how does this relate to the primary research question or risk of injury? Further, was the peak heart rate greater in females vs. males?

*Answer: As we wrote in revised text the main goals of the present study were to analyse: 1) the fitness parameters of Italian Firefighters, comparing males and females, 2) the impact of overload, represented by the protective garments, on the reaction time to the stimulus. We didn’t talk about heart rate reserve and about injury. We analysed the sex differences in function of job performance considering that lower cardiovascular fitness may induce higher fatigue that may reduce alertness and result in poor job performance and could also lead to an injury. Heart rate recovery is commonly used as an indicator of cardiovascular fitness (Shetler et al., 2001). So, high decrease of Heart Rate in short time after hard exercise is an index of good fitness.*

*To increase the clarity of text we changed as follow:*

*“…………….* anthropometric and fitness values between male and female Italian firefighter recruits. This study could give useful information to develop appropriate conditioning programmes aimed to reach an optimal job performance of firefighters. In fact, while much has been published…….”
